# The complete mitochondrial genome of dark-sided flycatcher *Muscicapa sibirica* (Passeriformes: Muscicapidae)

**DOI:** 10.1080/23802359.2019.1644240

**Published:** 2019-07-22

**Authors:** Cai-Hong Lu, Cheng-He Sun, Sen-Lin Hou, Ya-Lin Huang, Chang-Hu Lu

**Affiliations:** a College of Biology and the Environment, Nanjing Forestry University, Nanjing, China;; b Nanjing Forest Police College, Key Laboratory of State Forest and Grassland Administration on Wildlife Evidence Technology, Nanjing, China

**Keywords:** *Muscicapa sibirica*, mitochondrial genome, dark-sided flycatcher, *Muscicapa*

## Abstract

We report the mitochondrial genome of *Muscicapa sibiric*a. The overall base composition of the dark-sided flycatcher mitogenome is 24% T, 31.8% C, 29.4% A, and 14.8% G, with an A + T content of 53.4%. The total length of the sequence is 17,879 bp (13 protein-coding genes, 22 transfer RNA genes, 2 ribosomal RNA genes, and 2 control regions). Phylogenetic analysis was performed based on the concatenated nucleotide sequences of cytochrome c oxidase subunit I and cytochrome b using the neighbor-joining method and the Kimura 2-parameter model in MEGA 7.0 with 1000 bootstrap replicates.

The dark-sided flycatcher is a kind of passerine in the Muscicapidae family that breeds mainly in Northeast Asia and the Himalayas and migrates to Southern China, Palawan Island, Southeast Asia, and the Greater Sunda Islands in winter. There are three subspecies included in *Muscicapa sibirica*, namely, *M. sibirica cacabata*, *M. sibirica rothschildi*, and *M. sibirica sibirica. Muscicapa sibirica* is a summer-migratory bird in China, some of which survive the winter. From the end of April to the beginning of May, it arrives in the northeastern breeding ground and moves from the breeding ground to the wintering ground in September-October. It lives in arboreal habitats and feeds mainly on insects and insect larvae.

This study reports, for the first time, the complete mitochondrial genome (mtDNA) sequence of *M. sibirica*. Samples were collected at the bird circle station of the Jiangsu Dafeng Milu National Natural Reserve (33°05′N, 120°49′E) in October 2018 and after sampling, the specimens (NJFU-201803) were stored in the animal specimens museum of Nanjing Forestry University.

The mitogenome of *M. sibirica* is a closed, circular molecule composed of 17,879 bp (GenBank accession no. MK770601). The nucleotide composition is 24% T, 31.8% C, 29.4% A, and 14.8% G, with an A + T content of 53.4%. There are 22 transfer RNA genes (tRNA), 13 protein-coding genes (PCGs), 2 ribosomal RNA genes (rRNA), and 2 control regions in the mitogenome and it has a typical, vertebrate mitochondrial gene arrangement (Liu et al. 2019; Sun et al. 2019; Zeng et al. 2019).

In this study, phylogenetic analysis of *M. sibirica* and 13 other birds was carried out based on the concatenated nucleotide sequences of cytochrome c oxidase subunit I (COI) and cytochrome b (Cyt b), using the neighbor-joining method and the Kimura 2-parameter model in MEGA 7.0, with 1000 bootstrap replicates (Kumar et al. 2016).

The mitogenome of *M. sibirica* was genetically closest to that of *Ficedula zanthopygia* (Figure 1), which is in accordance with the current morphological classification and can, therefore, contribute to our understanding of the phylogeny and evolution of *Ficedula* and *Muscicapa* (Boore 1999; Sun et al. 2016; Caparroz et al. 2018).

**Figure 1.**
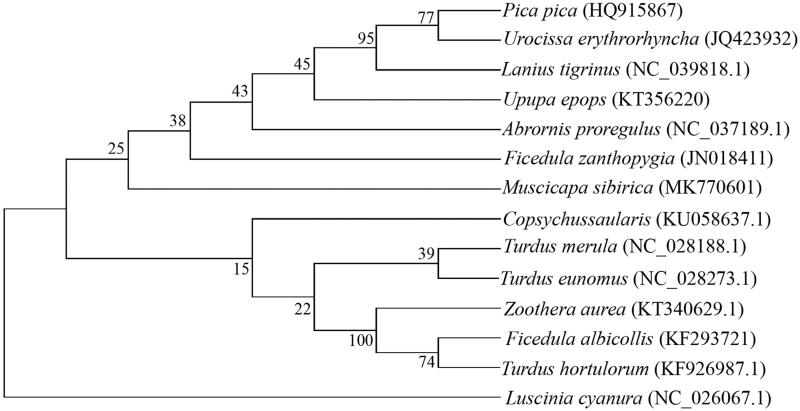
Neighbor-joining phylogenetic tree based on the concatenated nucleotide sequences of cytochrome c oxidase subunit I and cytochrome of *M. sibirica* and 13 other birds using MEGA 7.0.
